# Thermokinetic Parameters and Shelf-Life Prediction of Sacha Inchi Oil Stabilized with Essential Oils

**DOI:** 10.3390/molecules31193574

**Published:** 2026-10-08

**Authors:** María Alina Cueva-Ríos, Frank Fernandez-Rosillo, Javier S. Córdova-Ramos, Beetthssy Z. Hurtado-Soria, Eudes Villanueva, Gilbert Rodríguez, Elza Aguirre

**Affiliations:** 1 Departamento de Ingeniería de Industrias Alimentarias, Universidad Nacional de Jaén (UNJ), Cajamarca 06801, Peru; frank_fernandez@unj.edu.pe; 2 Escuela Profesional de Ciencia de los Alimentos, Universidad Nacional Mayor de San Marcos, Lima 09001, Peru; jcordovar1@unmsm.edu.pe; 3 Departamento Académico de Ingeniería en Industrias Alimentarias, Universidad Nacional Autónoma de Tayacaja Daniel Hernández Morillo (UNAT), Urb. Av. Universitaria s/n, Ahuaycha 09151, Peru; beetthssy.hurtado@unat.edu.pe (B.Z.H.-S.); eudesvillanueva@unat.edu.pe (E.V.); 4 Facultad de Ingeniería, Universidad Nacional del Santa (UNS), Urb. Av. Universitaria s/n, Nuevo Chimbote 02712, Peru; grodriguez@uns.edu.pe; 5 Laboratorio de Microbiología y Toxicología de Productos Agroindustriales, Universidad Nacional del Santa (UNS), Urb. Av. Universitaria s/n, Nuevo Chimbote 02712, Peru; eaguirre@uns.edu.pe

**Keywords:** *Plukenetia volubilis* L., lipid peroxidation, radical scavenging, reaction energetics, accelerated testing, lipid matrix, natural extracts

## Abstract

Sacha inchi oil is a highly unsaturated edible oil with considerable nutritional value but high susceptibility to oxidative deterioration. This study evaluated the effects of matico (*Piper aduncum* L.), real lemon (*Citrus limon* (L.) Burm. f.), and spearmint (*Mentha spicata* L.) essential oils (EOs) on the thermokinetic behavior, oxidative stability, and estimated shelf-life of sacha inchi oil. EOs were incorporated at 0.1%, 0.3%, and 0.5% (*v v*^−1^), and oxidative stability was evaluated by Rancimat at 90, 100, and 110 °C after 0, 7, 15, 30, and 90 days of storage. Activation energy (Ea), temperature coefficient (Q_10_), and changes in activation enthalpy (ΔH^++^), activation entropy (ΔS^++^), and Gibbs free energy (ΔG^++^) were calculated from the oxidative stability index (OSI) and used to estimate the shelf-life of the oil at storage temperature (T = 25 °C). EO type, EO concentration, and storage time had significant effects on the estimated shelf-life of sacha inchi oil (*p* < 0.05). Spearmint showed the most persistent protective effect, with the highest OSI values and an estimated shelf-life of approximately 350 days, whereas real lemon exhibited a more consistent protective effect throughout storage. EOs increased the activation energy (Ea) from 113.22 kJ mol^−1^ in the control to approximately 120 kJ mol^−1^ in the treated samples, indicating a higher apparent energetic barrier to oxidation. The temperature coefficient (Q_10_) increased from 2.665 to approximately 2.86, while the activation enthalpy (ΔH^++^) and Gibbs free energy (ΔG^++^) also increased in EO-treated samples, and the positive activation entropy (ΔS^++^) values indicated greater molecular disorder in the transition state. Overall, essential oils modulated the thermokinetic behavior of sacha inchi oil, increasing the apparent energetic barrier to oxidation and extending its predicted shelf-life, highlighting their potential as natural systems for stabilizing highly unsaturated edible oils.

## 1. Introduction

Sacha inchi (*Plukenetia volubilis* L.) is a lipid-rich seed valued for its high content of polyunsaturated fatty acids (PUFAs) [1]. Sacha inchi oil is recognized as a valuable functional oil due to its exceptional content of polyunsaturated fatty acids (PUFAs), particularly α-linolenic acid (ω-3), which accounts for approximately 85% of its lipid fraction [2]. In addition, the oil exhibits a favorable ω-3/ω-6 ratio [3], contributing to its nutritional value and potential health benefits [4,5,6]. Owing to these nutritional properties, sacha inchi oil has attracted increasing interest as a functional food ingredient [1,7,8]. However, the same high concentration of PUFAS that confers its nutritional value also increases its susceptibility to oxidative deterioration, compromising quality, sensory attributes, and shelf-life [9]. Therefore, maintaining oxidative stability throughout processing and storage is essential to preserve the integrity of PUFAs and minimize lipid oxidation [10].

Lipid oxidation is a major factor limiting the quality and shelf-life of edible oils [11]. Rancidity may result from thermo-oxidative and autoxidative processes, and the rate of oxidation is influenced by fatty-acid composition and environmental factors such as temperature and oxygen exposure [6,12]. Lipid oxidation can generate secondary oxidation products and cause undesirable changes in the chemical, physical, and sensory properties of edible oils [2,13]. Oxidative stability is therefore influenced by factors including temperature, light, oxygen exposure, extraction conditions, and antioxidant composition [4,14]. In general, greater oxidative stability is associated with greater resistance to lipid oxidation [15], and the incorporation of antioxidants can improve the oxidative stability of edible oils [16].

Antioxidants can delay lipid oxidation by interfering with radical-mediated oxidation pathways [17]. Many natural antioxidants contain phenolic structures, including tocopherols, flavonoids, and phenolic acids [18]. Natural antioxidant sources include plants and essential oils, and their effectiveness can be evaluated through accelerated oxidation methods such as the Rancimat test [15]. Improving oxidative stability through natural antioxidants may therefore contribute to extending shelf-life and preserving the quality of edible oils.

Essential oils are aromatic complex chemical mixtures composed mainly of aldehydes, phenols, ketones, esters, alcohols, and terpenes of commercial interest [19,20]. In addition to these compounds, EOs exhibit a wide range of biological activities, including antioxidant properties, largely associated with terpenoids that contribute to human health [21,22]. Their chemical composition varies considerably among plant species, and differences in terpene, phenolic, and other volatile constituents may influence their effectiveness in stabilizing highly unsaturated edible oils.

The essential oils selected in this study (*Piper aduncum* L., *Citrus limon* (L.) Burm. f., and *Mentha spicata* L.) were chosen because of their distinct phytochemical profiles and reported antioxidant properties. *P. aduncum* essential oil contains phenylpropanoids and terpenoid compounds and exhibits radical-scavenging activity [23], whereas *C. limon* essential oil is rich in monoterpenes, particularly limonene, and has demonstrated antioxidant activity [24]. *M. spicata* essential oil also exhibits antioxidant activity associated with its diverse volatile composition [25]. These chemically distinct essential oils therefore provide suitable systems for investigating how different antioxidant profiles influence the oxidative stability and thermokinetic behavior of sacha inchi oil.

Although oxidative stability is commonly evaluated using the oxidative stability index (OSI), thermokinetic analysis provides a deeper understanding of the oxidation mechanism by describing the energy requirements governing lipid degradation. Parameters such as activation energy (Ea), change in the activation enthalpy (ΔH^++^), activation entropy (ΔS^++^), Gibbs free energy (ΔG^++^), and the temperature coefficient (Q10) provide valuable information regarding the resistance of lipid systems to oxidation and allow estimated shelf-life under different storage conditions [26].

Previous studies have demonstrated the potential of essential oils to improve the oxidative stability and estimated shelf-life of sacha inchi oil. Luna-Fox et al. [27] reported that the incorporation of essential oils increased the oxidative stability and estimated shelf-life of sacha inchi oil, including the use of *Citrus limon* essential oil. Similarly, Cueva-Rios and Vargas [28] previously evaluated the oxidative stability, sensory quality, and estimated shelf-life of sacha inchi oil supplemented with essential oils from aromatic species from Jaén, Peru. Thus, the capacity of essential oils to improve oxidative stability and estimated shelf-life in sacha inchi oil has already been reported. However, these previous studies did not determine thermokinetic parameters describing the energetic and thermodynamic characteristics of the oxidation process.

Although phenolic constituents are often associated with antioxidant capacity, several studies have shown that the antioxidant activity of essential oils is not exclusively attributed to phenolic compounds but also to terpene structures with phenolic or non-phenolic characteristics [29]. These compounds may act through mechanisms such as chain-breaking and termination-enhancing reactions, interrupting the propagation phase of lipid oxidation and improving oxidative stability, while terpenes such as pinenes and other monoterpenes have demonstrated the ability to act as effective antioxidants in lipid systems [30]. Consequently, essential oils can serve as natural antioxidant sources capable of enhancing the oxidative stability of edible oils. The antioxidant activity of essential oils may therefore arise from the combined action of multiple constituents, including phenolic and non-phenolic terpenes, which can interfere with the propagation and termination stages of lipid oxidation [29,30]. Because the effectiveness of an essential oil depends on its chemical composition and on interactions among its constituents, different essential oils may provide different degrees and persistence of oxidative protection.

The knowledge gap addressed in the present study is therefore the lack of thermokinetic characterization of sacha inchi oil treated with essential oils. In particular, limited information is available on how essential oil type, concentration, and storage time influence apparent activation energy (Ea), temperature coefficient ($Q_{10}$), activation enthalpy (ΔH^++^), activation entropy (ΔS^++^), and Gibbs free energy (ΔG^++^). Determining these parameters provides complementary information to conventional oxidative stability and shelf-life measurements by describing the apparent energetic and thermodynamic behavior of the oxidation process.

Accordingly, the novelty of the present study lies in the thermokinetic characterization of sacha inchi oil treated with different essential oil types and concentrations and evaluated over different storage periods, integrating these parameters with oxidative stability and estimated shelf-life. Rather than establishing again that essential oils can extend shelf-life, which has already been reported [27,28], the present study characterizes the energetic and thermodynamic aspects underlying oxidative deterioration and examines how these characteristics vary according to essential oil treatment and storage time.

Therefore, this study aims to investigate how essential oil type, concentration, and storage time affect the thermokinetic behavior, oxidative stability, and estimated shelf-life of sacha inchi (*Plukenetia volubilis* L.) oil. Specifically, the apparent activation energy, temperature coefficient, and activation thermodynamic parameters (enthalpy, entropy, and Gibbs free energy) were determined to characterize the apparent energetic and thermodynamic behavior of oxidative deterioration and to examine their relationship with the estimated shelf-life of sacha inchi oil under the different essential oil treatments.

## 2. Results and Discussion

### 2.1. Physicochemical and Antioxidant Characterization of Sacha Inchi Oil and Essential Oils

Sacha inchi oil has been extensively studied for its nutritional profile and potential as a functional food. In the data analyzed (Table 1), this oil, which represented 45.33% of the seed, demonstrated a lipid composition that is particularly beneficial to human health. It is noteworthy for its high content of PUFAS, which make up more than 81% of the total fatty acids. Specifically, alpha-linolenic acid (C18:3, ω-3) accounts for 46.7%, followed by linoleic acid (C18:2, ω-6) at 35.27%. The fatty acid profile obtained in the present study is consistent with that reported by Maurer et al. [31], particularly regarding the predominance of α-linolenic and linoleic acids, confirming the characteristic fatty acid composition of sacha inchi oil. This combination of essential fatty acids provides an excellent ω-6/ω-3 ratio of 0.755, which is very favorable for the human body, as an imbalance in this ratio, particularly an excess of ω-6, has been associated with inflammatory processes and cardiovascular diseases [32,33]. On the other hand, the oil contains a low proportion of SFA, totaling 7.34%, with palmitic acid (C16:0) being the predominant one. Likewise, MUFAS account for only 9.75%, with oleic acid (C18:1) being the most abundant in this category. This distribution confirms the unsaturated and cardioprotective nature of sacha inchi oil, as has already been reported in previous research [34,35,36].

Regarding its physicochemical properties, quality indicators such as the peroxide value (4.89 mEqO_2_ kg^−1^) and the acidity index (0.43 mg KOH g^−1^) are below the maximum limits established by the Codex Alimentarius [37], indicating good oxidative stability of the oil in its current state. Moreover, the iodine value (178.12 mg I_2_ g^−1^) reflects the high degree of unsaturation of the oil, confirming its richness in essential fatty acids. The p-anisidine value (15.26) and the TotOx index (24.99) complement this assessment, as they help estimate the secondary and total oxidation of the oil. Both values indicate a product that is still fresh and suitable for consumption, although subject to change depending on storage conditions [38]. In terms of bioactive compounds, this oil presents a significant content of total phenolic compounds (TPC), with a value of 110.23 mg GAE/100 g of oil. This amount is comparable to, and even higher than, that of vegetable oils renowned for their functional value, such as extra virgin olive oil, whose typical values range from 50 to 100 mg GAE 100 g^−1^ [39]. These phenolic compounds play a key antioxidant role, protecting lipids from oxidation and contributing to the oil’s stability. Finally, antioxidant capacity (AC), measured in this study as 350.65 µmol Trolox g^−1^, is remarkably high. This value reflects the oil’s ability to neutralize free radicals and prevent oxidative processes, both in food matrices and within the human body. Compared to other vegetable oils, this figure positions sacha inchi oil among the most active in terms of antioxidant potential [40,41]. In summary, the characterization of sacha inchi oil demonstrates that this product possesses an ideal lipid profile, good physicochemical stability, and a high content of bioactive compounds.

The physicochemical characterization of essential oils extracted from matico, real lemon, and spearmint leaves showed significant differences (*p* < 0.05) in extraction yield, antioxidant capacity (AC), and volatile compound profile (Table 2). These differences could be attributed to the specific anatomical properties of the leaves, the density and type of glandular trichomes, as well as the chemical composition of each species. The extraction yield by steam distillation was variable. The highest yield was obtained in real lemon leaves (0.530%), which is relevant, as citrus essential oils are usually derived from the fruit pericarp [42]. Nevertheless, this result suggests a considerable presence of volatile metabolites in real lemon leaves, possibly associated with specialized epidermal structures. The lowest yield was recorded in matico leaves, in agreement with studies reporting lower essential oil content in leaves than in inflorescences of this species [43]. Spearmint presented an intermediate yield, which is consistent with the literature on leaf essential oils of the genus *Mentha* [44].

Antioxidant capacity (AC) differed significantly among the essential oils. Matico exhibited the highest antioxidant capacity (7023.188 µmol Trolox g^−1^), followed by real lemon and spearmint. This higher antioxidant activity may be associated with the presence of its major volatile constituents, particularly the phenylpropanoids dillapiole (352.10 mg g^−1^) and myristicin (145.05 mg g^−1^), which have been reported to possess free radical scavenging and redox-active properties [45]. Likewise, synergistic interactions among the different volatile constituents may also contribute to the overall antioxidant activity of the essential oil, as previously reported for aromatic plant extracts [46].

The selected volatile compounds quantified in the present study showed differences among the essential oils. In real lemon essential oil, limonene presented the highest concentration among the quantified compounds (428.42 mg g^−1^). This monoterpene has been associated with antimicrobial and antioxidant properties in previous studies [47]. In spearmint essential oil, menthol and menthone were the compounds selected for quantitative analysis. These oxygenated monoterpenes have been associated with characteristic sensory properties and biological activities [48]. In matico essential oil, the quantified compounds corresponded to the phenylpropanoids dillapiole and myristicin, which have been previously reported as characteristic constituents of this species and have been associated with diverse biological activities [49]. The differences observed among the quantified compounds suggest that the essential oils may exhibit distinct functional properties according to their botanical origin.

### 2.2. Oxidative Stability Index and Estimated Shelf-Life

The oxidative stability index (OSI) is a key parameter for evaluating the resistance of vegetable oils to oxidation. In the present study, the effect of three essential oils (EOs) (matico, real lemon, and spearmint) on the OSI of sacha inchi oil was examined, considering three concentrations (0.1%, 0.3%, and 0.5%), five storage periods (0, 7, 15, 30, and 90 days), and three temperatures (90 °C, 100 °C, and 110 °C). These OSI measurements represent oxidative stability under the respective accelerated oxidation conditions (Rancimat) and should not be interpreted as direct measurements of shelf-life under real storage conditions.

As shown in Table 3, the addition of EOs significantly increased the OSI compared to the control, confirming the antioxidant contribution of these natural extracts. At 90 °C, all three EOs improved the oxidative stability of the oil compared to the control (6.717 h). Among them, spearmint EO showed high OSI values, reaching OSI values of up to 8.537 h (0.1%, 7 days) and 8.653 ± 0.026 h (0.5%, 7 days). The superior performance of spearmint cannot be explained simply by its total antioxidant capacity measured by DPPH, since spearmint showed the lowest DPPH activity among the three EOs (777.429 µmol Trolox g^−1^), whereas matico showed the highest value (7023.188 µmol Trolox g^−1^) (Table 2). This apparent discrepancy suggests that the antioxidant behavior of an essential oil in a lipophilic matrix depends not only on its radical-scavenging capacity in a chemical assay, but also on the chemical nature, polarity, stability, and interactions of its constituents within the lipid matrix.

The GC–MS characterization showed that spearmint EO was mainly characterized by menthol (376.5 mg g^−1^), a monoterpene alcohol, and menthone (219.5 mg g^−1^), a monoterpene ketone. These oxygenated monoterpenes may contribute to the protection of the lipid matrix through interactions with lipid radicals and hydroperoxides and may provide a more persistent antioxidant contribution under the experimental conditions. Therefore, the higher OSI observed for spearmint may result from the combined effect of its major constituents rather than from a single compound. Previous studies have demonstrated that the antioxidant activity of essential oils depends strongly on their chemical composition and on the interactions among their constituents [50,51].

Real lemon EO also showed good results, although slightly lower. Its major identified constituents were limonene (428.4 mg g^−1^) and β-pinene (183.1 mg g^−1^), both monoterpene hydrocarbons. Although these compounds can contribute to the antioxidant behavior of citrus essential oils, their chemical structures differ from the oxygenated monoterpenes identified in spearmint. The lower OSI observed for real lemon may therefore reflect differences in the chemical composition and antioxidant mechanisms of the respective oils. In addition, the relatively volatile nature of monoterpene hydrocarbons may influence their persistence during prolonged storage and exposure to elevated temperatures.

In contrast, matico EO, while improving OSI compared to the control, demonstrated lower antioxidant efficacy and a more pronounced tendency to decline over time. Interestingly, matico exhibited the highest DPPH antioxidant capacity (7023.188 ± 6.325 µmol Trolox g^−1^), despite showing lower persistence in the OSI measurements. Its major constituents were dillapiole (352.1 mg g^−1^) and myristicin (145.1 mg g^−1^), both classified as phenylpropanoids. This difference between DPPH activity and oxidative stability indicates that a high radical-scavenging response in a chemical assay does not necessarily translate into sustained protection of a highly unsaturated lipid matrix. The progressive reduction in OSI observed for matico may be associated with differences in the stability, partitioning, or interactions of its phenylpropanoid constituents under prolonged thermal and oxidative conditions. In particular, prolonged exposure to temperatures of 90–110 °C under oxidative conditions, as used in the Rancimat assay, could potentially promote the transformation or degradation of certain phenylpropanoids, thereby reducing their effective concentration and protective contribution during lipid oxidation. However, the thermal degradation of dillapiole and myristicin was not directly evaluated in the present study; therefore, this explanation remains hypothetical. Further investigations involving the monitoring of individual phenylpropanoids during thermal exposure and the evaluation of their antioxidant activity before and after heating would be required to confirm this mechanism. Thus, the apparent discrepancy between the high DPPH activity and the lower persistence of matico in the OSI measurements may reflect differences between radical-scavenging capacity under the DPPH assay conditions and the persistence of antioxidant protection in a highly unsaturated lipid matrix.

At 100 °C, the antioxidant effect of the EOs was maintained, albeit with a slight decrease in OSI values due to the temperature increase, which aligns with the accelerated lipid peroxidation processes at higher temperatures [52]. Once again, spearmint EO proved highly effective, reaching 3.130 h (0.5%, day 0), while real lemon and matico EOs recorded values of 3.073 h and 3.593 h, respectively. The persistence of the protective effect of spearmint under elevated temperature suggests that its overall chemical profile provided an effective barrier against oxidation under accelerated conditions. In contrast, the decline observed for matico with storage may indicate a lower persistence of the antioxidant contribution of its major constituents under prolonged exposure to thermal and oxidative stress.

Under more severe conditions (110 °C), OSI values were naturally lower across all treatments. Nonetheless, the EOs continued to provide protection against oxidation. Spearmint EO at 0.5% maintained the highest OSI at this temperature (1.317 h), followed by real lemon (1.247 h) and matico (1.171 h). The fact that spearmint maintained the highest OSI under the most severe temperature condition further supports the importance of the chemical composition of its constituents. In particular, its major compounds, menthol and menthone, are oxygenated monoterpenes characteristic of spearmint essential oil and have been associated with antioxidant activity [53]. However, the present results do not demonstrate that menthol or menthone alone are responsible for this effect or that they are individually more thermally stable than the constituents of the other essential oils. Rather, the observed protection should be interpreted as the result of the combined chemical profile of the complete essential oil and the possible interactions among its constituents.

Overall, the results indicate that the antioxidant effectiveness of the EOs cannot be predicted solely from their DPPH activity. Matico showed the highest DPPH activity but did not provide the most persistent oxidative protection, whereas spearmint showed the lowest DPPH activity but the highest OSI under the evaluated conditions. This finding highlights the importance of considering the chemical composition, physicochemical behavior, concentration, and matrix interactions of essential oils when evaluating their protective effects in highly unsaturated edible oils. Possible synergistic or antagonistic interactions among individual constituents may also contribute to the observed differences; however, these interactions were not directly evaluated in the present study and therefore should be considered as possible rather than demonstrated mechanisms.

A comparison of the different EO concentrations revealed a general tendency toward greater oxidative stability at higher concentrations, although this effect depended on the EO type and storage conditions. This concentration-dependent behavior has been reported for essential oils, whose antioxidant effects may increase with concentration until a saturation point is reached [54]. In the present study, the effect was particularly evident for spearmint, which maintained the highest OSI at 110 °C at 0.5% EO (1.317 h), whereas real lemon and matico showed lower values of 1.247 and 1.171 h, respectively. Importantly, the response during storage was not identical among EOs.

Spearmint and real lemon showed relatively stable protection, whereas matico exhibited a progressive reduction in oxidative stability, suggesting that the persistence of the antioxidant effect depends on the chemical composition and stability of the individual EO constituents. These results are consistent with previous research showing that essential oils can improve the oxidative stability of lipid matrices such as sacha inchi oil [28]. Quantitatively, Rodríguez et al. [33] reported an activation energy of 115.13 ± 1.64 kJ mol^−1^ for sacha inchi oil, whereas the control in the present study showed 113.22 kJ mol^−1^ and EO-treated samples reached approximately 121 kJ mol^−1^. Thus, EO incorporation increased the apparent activation energy by approximately 6.9% relative to the control, supporting an increase in the energetic barrier to oxidation. Similarly, the $Q_{10}$ values observed in the present study (2.665—approximately 2.86) were comparable to the value previously reported for sacha inchi oil (2.62 ± 0.43) [33].

Wang et al. [4] also demonstrated that natural antioxidants can improve the oxidative stability of oils rich in polyunsaturated fatty acids, including sacha inchi oil. However, the present results further indicate that the magnitude and persistence of this protection depend on EO composition, concentration, and storage conditions. Therefore, the antioxidant effect should not be attributed solely to the total radical-scavenging capacity measured by DPPH, but rather to the combined chemical behavior of the EO constituents within the lipid matrix. Essential oils contain diverse bioactive compounds, including terpenoids and phenylpropanoids, which may interfere with lipid-radical propagation and thereby delay oxidative deterioration [55].

Recent reviews have further emphasized that the oxidative stability of ω-3-rich oils depends not only on the intrinsic antioxidant capacity of the added compounds, but also on their chemical composition, lipophilicity, stability, interactions, and behavior within the lipid matrix. Natural antioxidants have shown considerable potential for protecting highly unsaturated oils such as sacha inchi, chia, and flaxseed oils; however, their effectiveness may vary depending on the antioxidant system, concentration, and processing or storage conditions [56,57,58]. These considerations are particularly relevant to the present results, as the differences in oxidative protection among spearmint, real lemon, and matico essential oils were not proportional to their DPPH antioxidant capacity, suggesting that the chemical composition and behavior of each essential oil within the lipid matrix played an important role in determining its protective effect.

Regarding shelf-life projection, the results (Table 3) showed that the addition of essential oils significantly increased the estimated shelf-life of sacha inchi oil compared with the control (*p* < 0.05). The control exhibited the shortest estimated shelf-life (~170 days), reflecting the high susceptibility of this highly unsaturated oil to oxidative deterioration. This observation is consistent with previous reports describing sacha inchi oil as a lipid-rich matrix with a high proportion of polyunsaturated fatty acids, particularly α-linolenic acid, which contributes to its oxidative susceptibility [4].

Matico EO showed a moderate protective effect, with an initial increase in estimated shelf-life (~290 days), particularly during the first 15 days, followed by a decreasing trend toward day 90. This behavior may be associated with changes in the availability or stability of volatile and bioactive constituents during prolonged storage. Although the estimated shelf-life remained significantly higher than that of the control (*p* < 0.05), the progressive reduction observed over storage suggests a possible decrease in antioxidant effectiveness over time.

In contrast, real lemon EO showed a more consistent effect. Throughout the 90-day storage period, the estimated shelf-life remained within approximately 300–310 days, with relatively small variations. This stability may be associated with the presence of bioactive monoterpenes and other oxygenated compounds reported in citrus essential oils, which have been associated with antioxidant activity [54]. However, the sustained shelf-life observed in the present study should be interpreted as an experimental observation rather than being attributed exclusively to individual compounds, since the antioxidant behavior of essential oils depends on their overall chemical composition and possible interactions among constituents.

Finally, spearmint EO exhibited the greatest protective effect. From the beginning of the experiment to day 90, the estimated shelf-life remained at approximately 350 days, indicating a sustained protective effect against oxidation. This behavior may be related, at least in part, to the chemical composition of spearmint essential oil, particularly to the oxygenated monoterpenes identified and quantified in the present GC–MS analysis, including menthol and menthone. These compounds, together with other constituents of the complete essential oil profile, may have contributed to the observed oxidative stability [50]. The absence of an evident decrease in estimated shelf-life during storage suggests that spearmint essential oil provided relatively stable protection under the conditions evaluated.

Overall, these results are consistent with previous studies demonstrating that essential oils can improve the oxidative stability of lipid matrices. However, their effectiveness depends not only on the type of essential oil but also on its concentration, chemical composition, interactions among constituents, and storage conditions. From a practical perspective, spearmint EO showed the greatest potential for extending the estimated shelf-life of sacha inchi oil, followed by real lemon EO, whereas matico EO provided a lower and less persistent protective effect. Further studies evaluating combinations of essential oils or their major constituents could help determine synergistic interactions and improve the stability of the antioxidant effect during prolonged storage.

The three-way factorial analysis presented in Table 4 allowed the simultaneous evaluation of the effects of essential oil (EO) type, EO concentration, and storage time on the estimated shelf-life of sacha inchi oil, as well as the possible interactions among these factors. The results showed that the three main factors had statistically significant effects on estimated shelf-life. EO type showed a significant effect (*p* = 0.0478; η^2^p = 0.0653), whereas EO concentration (*p* < 0.001; η^2^p = 0.6175) and storage time (*p* < 0.001; η^2^p = 0.6454) exhibited effects of greater magnitude.

In particular, the η^2^p values indicate that EO concentration and storage time accounted for a considerable proportion of the variability observed in estimated shelf-life. This suggests that, under the conditions evaluated, the amount of EO incorporated and the time elapsed during storage were important factors in explaining differences in estimated oxidative stability. The effect of EO type was statistically significant, although its relative magnitude was lower than that observed for EO concentration and storage time.

On the other hand, none of the evaluated interactions reached statistical significance. The EO × concentration interaction yielded *p* = 0.0761, whereas EO × storage time, concentration × storage time, and EO × concentration × storage time yielded p-values of 0.7361, 0.7671, and 0.9925, respectively. These results indicate that, under the experimental conditions studied, there was insufficient statistical evidence to conclude that the effect of one factor depended on the level of another factor. For example, although different EO concentrations significantly affected estimated shelf-life, this effect did not vary significantly according to EO type or storage time.

Overall, Table 4 shows that estimated shelf-life was influenced mainly by the individual effects of EO type, EO concentration, and storage time, with a particularly important contribution from concentration and storage time. The absence of significant interactions also indicates that the main effects can be interpreted independently within the experimental range evaluated. Nevertheless, these results correspond to an estimated shelf-life derived from accelerated oxidation conditions and should therefore not be interpreted as validation of the actual shelf-life of the oil under conventional storage conditions. Confirmation of these estimates would require real-time storage studies and monitoring of oxidation indicators over extended storage periods.

### 2.3. Thermokinetic Parameters

The thermokinetic parameters (Table 5) derived from the oxidative stability index (OSI) provide insight into the mechanisms controlling lipid oxidation in sacha inchi oil and the protective effect of essential oils (EOs). In this study, the activation energy (Ea), temperature coefficient (Q_10_), activation enthalpy (${\Delta H}^{++}$), activation entropy (${\Delta S}^{++}$), and Gibbs free energy of activation (${\Delta G}^{++}$) were evaluated. These parameters describe the energetic requirements and molecular changes associated with the oxidative degradation of PUFAS. Activation energy (Ea) is a key indicator of the susceptibility of oils to oxidation [52]. The control oil showed the lowest Ea value (113.22 kJ mol^−1^), whereas oils containing EOs reached values close to 121 kJ mol^−1^. The Ea value of the control was close to the 115.13 ± 1.64 kJ mol^−1^ previously reported for sacha inchi oil [33]. In contrast, EO-treated samples showed Ea values approximately 6.9% higher than the control, indicating an increase in the apparent energetic barrier associated with the overall oxidation process. The Ea values obtained were also within the range reported for highly unsaturated vegetable oils, including flaxseed and chia [59]. The higher Ea values observed in the EO-treated oils were consistent with their greater oxidative resistance. However, Ea should be regarded as an apparent energetic parameter reflecting the temperature dependence of the overall oxidation process, rather than as evidence of direct inhibition of a specific oxidation step. The higher Ea values, particularly for spearmint, suggest that its overall chemical composition may have increased the apparent energy barrier to lipid oxidation more effectively than matico and real lemon. This interpretation is consistent with the dependence of essential oil antioxidant activity on chemical composition and constituent interactions [52,54,60]. The $Q_{10}$ coefficient, which expresses the increase in reaction rate for every 10 °C rise in temperature, ranged from 2.665 in the control to approximately 2.86 in oils with EOs. These values, typical of lipid oxidation reactions, indicate a strong temperature dependence [32,61]. Although antioxidants delay oxidation, the process remains sensitive to temperature, highlighting the importance of thermal control during storage. Similar values have been reported in oils enriched with natural antioxidants such as rosemary extracts and phenolic compounds [58]. The change in the activation enthalpy values (${\Delta H}^{++}$) ranged approximately between 110 and 118 kJ mol^−1^, being higher in treatments containing EOs. This increase indicates that the formation of the transition state requires greater energy, reflecting the ability of natural antioxidants to interrupt the propagation of radical reactions by donating hydrogen atoms to lipid radicals [29]. Similar results have been observed in vegetable oils enriched with natural antioxidants [58,59]. On the other hand, the change in the activation entropy values (${\Delta S}^{++}$) was positive in all treatments (40.87–61.73 J mol^−1^ K^−1^), indicating an increase in molecular disorder in the transition state, which is a typical feature of free-radical reactions such as lipid peroxidation [38]. The higher ${\Delta S}^{++}$ values observed in oils with greater concentrations of essential oils may reflect interactions between antioxidant compounds and lipid radicals, generating intermediate complexes with greater molecular freedom [29,58]. Finally, the change in the Gibbs free energy (${\Delta G}^{++}$) ranged from 94.89 to 95.81 kJ mol^−1^, with slightly higher values in oils containing essential oils. This increase indicates that oxidation becomes thermodynamically less favorable, resulting in greater oxidative stability. Even small variations in ${\Delta G}^{++}$ can significantly influence the oxidation rate due to the exponential relationship described by transition state theory [59,60]. Overall, essential oils increased the apparent energetic parameters and oxidative stability under accelerated Rancimat conditions. These parameters reflect the temperature dependence of oxidation and should not be directly equated with real-time shelf-life under ambient storage. The treatment with 0.5% EOs generally exhibited the highest stability, supporting the potential of essential oils as natural antioxidants in edible oils and in the development of functional foods with a clean-label approach [62].

## 3. Materials and Methods

### 3.1. Raw Materials

Sacha inchi seeds were collected in the San Martin region, Lamas province (06°25′19″ S; 76°30′58″ W), at an altitude of 808 m.a.s.l. Subsequently, the seeds were subjected to an oven-drying process (SW-17TC-1, Blue M, New Columbia, PA, USA) until reaching a moisture content of 9.1 ± 0.12 g 100 g^−1^, in order to preserve the integrity of the lipid compounds. Oil extraction was performed by cold pressing, using a single screw expeller (CA 59 G Komet, IBG Monforts Oekotec GmbH & Co. KG, Mönchengladbach, Germany) at a speed of 35–40 rpm and maximum temperature of 50 °C. The oil was clarified by centrifugation and stored in dark jars at 4.0 ± 0.5 °C under a nitrogen atmosphere to prevent oxidation. On the other hand, fresh leaves of three plant species collected in the hamlet of Las Naranjas, district of Jaén, Cajamarca region (approximately 5°44′40.84″ S and 78°50′44.00″ W), in a premontane forest ecosystem, at altitudes between 1213 and 1256 m.a.s.l., were used. The first species, *Citrus limon* (L.) Burm. f., known as real lemon, corresponds to a tree up to 6 m tall, with simple, alternate, distichous leaves of intense green color. The second species, *Piper aduncum* L., commonly called matico, is a shrub approximately 3 m tall, with simple, alternate, oval or elliptical leaves, with an acuminate apex and rounded base. The leaves have a rough texture, with pubescence on the upper side and a marked ribbing. The third species corresponds to *Mentha spicata* L., known as spearmint. It is an herbaceous plant between 30 and 90 cm high, with lanceolate leaves, serrated margin, glabrous surface and hairy underside. It is characterized by its intense aroma, attributable to the presence of essential oils in its leaf tissue. The leaves of all species were subjected to a drying process at room temperature (28–29 °C) for 24 h, under shade and with adequate ventilation. The final humidity of the leaves was controlled until reaching a value of approximately 12%, determined with a humidity balance (Precisa Gravimetrics AG, LX320A, Port Townsend, WA, USA). The extraction of the essential oils was carried out by vapor entrainment, under controlled conditions of one atmosphere of pressure and temperature of 90 °C for 2 h.

### 3.2. Preparation of Oil Blends

Sacha inchi oil was used as the lipid matrix for the formulation of all experimental treatments. Essential oils (EOs) from matico, real lemon, and spearmint were directly incorporated into the oil at three concentration levels (0.1%, 0.3%, and 0.5%, *v v*^−1^), relative to the total volume of sacha inchi oil. The blending was performed without the use of solvents or emulsifying agents. The required volume of each essential oil was added directly to the sacha inchi oil under continuous magnetic stirring at 500 rpm for 10 min to ensure homogeneity. After mixing, the blends were immediately transferred into amber glass bottles, flushed with nitrogen gas to minimize oxidative reactions, and stored at 25 ± 0.5 °C until analysis. The control sample consisted of sacha inchi oil without the addition of essential oils. All formulations were prepared in triplicate.

### 3.3. Chemicals and Reagents

All chemicals and solvents used in this study were of analytical grade. Methanol (CH_3_OH), ethanol (C_2_H_5_OH), isooctane (C_8_H_18_), boron trifluoride (BF_3_, 14% in methanol), chloroform (CHCl_3_), and petroleum ether were supplied by Spectrum Chemical Mfg. Corp. (Spectrum^®^, Stamford, CT, USA). Folin–Ciocalteu reagent was obtained from Oxford Range of Laboratory Chemicals (Oxford^®^, New Delhi, India). 2,2-Diphenyl-1-picrylhydrazyl (DPPH) was purchased from HiMedia Laboratories Pvt. Ltd. (HiMedia^®^, Thane, Maharashtra, India). Gallic acid (C_7_H_6_O_5_) was supplied by Central Drug House (P) Ltd. (CDH^®^, New Delhi, India). Trolox, sodium thiosulfate (Na_2_S_2_O_3_), sodium carbonate (Na_2_CO_3_), sodium chloride (NaCl), potassium hydroxide (KOH), and sodium hydroxide (NaOH) were obtained from Merck KGaA (EMD Millipore Corp., Zug, Switzerland). High-purity hydrogen (99.9%), helium (99.9%), nitrogen (99.9%), and synthetic air (99.9%) used for chromatographic analyses were supplied by Air Products Perú S.A. (Experis^®^, Lima, Peru).

### 3.4. Physicochemical Characterization

To determine the extraction yield of the essential oils and sacha inchi oil, dried leaves of the selected plant species (real lemon, matico, and spearmint) and dehydrated sacha inchi seeds were used. The essential oils were extracted by steam distillation under controlled conditions using a stainless-steel distillation unit (internal diameter: 45 cm, external diameter: 54 cm, total height: 120 cm, chamber height: 80 cm, chamber volume: 50 L). The distillate was collected, and the oil phase was separated from the aqueous phase and stored in amber glass vials with airtight seals, protected from light and oxygen to prevent oxidative degradation. The extraction yield was calculated as the percentage by weight of the oil obtained relative to the initial dry mass of plant material, using the following formula:

$$\mathrm{Yield}\;(\%)\;=\;\frac{mass\;of\;extracted\;oil}{mass\;of\;dry\;plant\;material}\times 100$$

This value is expressed as a percentage by weight (*w w*^−1^) with respect to the initial dry weight of the processed biomass. In the case of sacha inchi oil, cold pressing was applied to the previously dried seeds, and the yield was calculated using the same formula.

The acid value (AV) was determined by the titration method defined in the official methods Cd 3d-63 of the American Oil Chemists’ Society [63]. Titration of the oil samples (10 g) dissolved in 50 mL of previously neutralized chloroform–ethanol medium (50:50 *v v*^−1^) was applied, using a 0.1 N potassium hydroxide (KOH) ethanolic solution as standard reagent to a phenolphthalein endpoint. AV was expressed as milligrams of KOH required to neutralize the free fatty acids present in 1 g of the oil sample (mg KOH g^−1^).

$$AV=\frac{G\times N\times 56.1}{w}$$

where *G* is the titratable volume of KOH (mL), *N* is the normality of KOH (0.1 N) and w is the weight of the sample (g).

The refractive index (RI) was measured according to method 921.08 [64] working at 25 °C and using a digital A 24051 refractometer (Rudolph Research Analytical, Hackettstown, NJ, USA) kept at 20 °C.

The iodine value (IV) was determined by the Wijs method, in accordance with method 993.20 [64], and 0.2 g of oil was dissolved with 10 mL of chloroform and 15 mL of Wijs reagent. After 45 min of rest, 10 mL of potassium iodide 15% was added in 50 mL of distilled water and proceeded to titrate with sodium thiosulfate (0.1 N) with a brown to yellow color change. A total of 1 mL of 1% soluble starch was added and titrated again with 0.1 N sodium thiosulfate until the color changed from blue to white. IV is expressed as mg I_2_ g^−1^.

$$IV=\frac{(B-M)\times N\times 12.65}{w}$$

where *B* and M are the titratable volumes of sodium thiosulfate (mL) for the blank and oil sample, respectively. *N* is the sodium thiosulfate normal (0.1 N), and w is the sample weight (g).

The peroxides value (PV) was determined following the Cd 8–53 method [63] with modifications, where 5 g of oil was dissolved in 30 mL of acetic acid–chloroform solution (60/40, *v v*^−1^). A total of 0.5 mL of saturated potassium iodide solution was added and allowed to stand for 1 min in the dark, then 30 mL of distilled water was added and stirred for 5 min. Finally, 0.5 mL of 1% starch solution was added and titrated with 0.01 N sodium thiosulfate solution. PV is expressed in milliequivalents of active oxygen present in 1 kg of oil (mEqO_2_ kg^−1^).

$$PV=\frac{G\times N\times 1000}{w}$$

where *G* is the titratable volume of sodium thiosulfate (mL), *N* is the normality of sodium thiosulfate (0.01 N) and w is the weight of the sample (g).

The p-anisidine (p-AV) value was monitored using the Cd 18–90 [63] with modifications. Two reagent dilutions were prepared, the first consisted of the oil/isooctane mixture (0.5 g 25 mL^−1^), and the second was anisidine reagent/acetic acid (0.025 g 25 mL^−1^). Subsequently, the absorbances of the dilutions were measured at 350 nm, according to the following description:

$$p\text{-}AV=\frac{25\;\times \;(1.2\;\times \;As\;-\;Ab)}{w}$$

where As is absorbance of oil/isooctane minus absorbance of pure isooctane. Ab is absorbance of the oil/isooctane diluted in anisidine/acetic acid (1/1, *v v*^−1^) minus the absorbance of the anisidine/acetic acid diluted in isooctane (1/1, *v v*^−1^). w is the weight of the oil (g). Finally, the total oxidation value (TotOx) has been determined as: TotOx = 2PV + p-AV.

### 3.5. Fatty Acid Composition

The fatty acid composition of sacha inchi oil was determined according to the fatty acid methyl ester (FAMES) method 991.39 [65]. Briefly, 0.025 g of oil was weighed and reacted with 1.5 mL of 0.5 N NaOH in a water bath (Foss, WB1024, Hillerød, Denmark) at 90 °C for 5 min. The reaction mixture was then cooled to approximately 30 °C, after which 2.0 mL of 14% boron trifluoride (BF_3_) was added in methanol. Subsequently, the reaction mixture was heated at 100 °C for 30 min, cooled to room temperature, and mixed with 1 mL of isooctane and 5 mL of saturated NaCl solution under continuous stirring while maintaining a nitrogen atmosphere. Identification of the fatty acid methyl esters was performed using a gas chromatograph (Shimadzu GC-2010, Kyoto, Japan) equipped with a flame ionization detector (FID) and a Shimadzu AOC-20Si autosampler. An SP-2560 fused-silica capillary column (100 m × 0.25 mm i.d., 0.20 μm film thickness) was used with helium as the carrier gas at a flow rate of 1.41 mL min^−1^ and a pressure of 261.5 kPa. The injection volume was 1.0 μL. The injector temperature was maintained at 225 °C (split mode), and the detector temperature was maintained at 250 °C. The oven temperature program consisted of an initial temperature of 100 °C held for 4 min, followed by an increase to 240 °C at a rate of 3 °C min^−1^ and a final hold of 10 min. Each sample was analyzed in triplicate.

### 3.6. Selected Predominant Volatile Compounds in Essential Oils

The essential oils of matico, real lemon, and spearmint were initially analyzed by gas chromatography coupled with mass spectrometry (GC–MS), following the methodology proposed by Adams [66], with minor modifications. Analyses were carried out using a Shimadzu GCMS-QP2020 system (Shimadzu Corporation, Kyoto, Japan) equipped with an AOC-20i autosampler and an Rxi^®^-5Sil MS fused-silica capillary column (30 m × 0.25 mm i.d., 0.25 μm film thickness; Restek, Bellefonte, PA, USA). Helium (99.999% purity) was used as the carrier gas at a constant flow rate of 1.0 mL min^−1^. Samples (1 μL) were injected in split mode (1:20), with the injector maintained at 250 °C. The oven temperature was initially maintained at 50 °C for 3 min, increased to 200 °C at 4 °C min^−1^, and subsequently increased to 250 °C at 10 °C min^−1^, where it was held for 5 min. The mass spectrometer operated under electron ionization (EI) at 70 eV, with the ion source and interface temperatures maintained at 230 °C and 250 °C, respectively. Mass spectra were acquired in full-scan mode over an *m*/*z* range of 40–500.

The initial GC–MS analysis was used to characterize the volatile profile of each essential oil and to identify the predominant volatile compounds. Tentative compound identification was performed by comparing the acquired mass spectra with reference spectra from the NIST (National Institute of Standards and Technology) Mass Spectral Library. Retention indices (RIs) were not determined because an n-alkane series was not analyzed under the same chromatographic conditions. Therefore, compound assignments based solely on mass spectral library comparison were regarded as tentative.

Based on the initial chromatographic profile, the predominant volatile compounds selected for subsequent quantitative analysis were dillapiole and myristicin in matico essential oil, limonene and β-pinene in real lemon essential oil, and menthol and menthone in spearmint essential oil. These selected compounds were subsequently quantified using certified analytical standards (≥98% purity; Sigma-Aldrich, Merck, Darmstadt, Germany). Calibration curves were prepared using standard solutions at different concentration levels according to the procedures described by Juncos et al. [30] and Olmedo et al. [14]. Linear regression was used to establish the relationship between peak area and analyte concentration, with coefficients of determination (R^2^) greater than 0.99. The resulting calibration equations were used to quantify the selected compounds in the essential oil samples, after accounting for the corresponding sample dilution factors. Each sample was analyzed in triplicate.

The concentrations obtained from the calibration curves were expressed as mg of analyte per g of essential oil (mg g^−1^ EO). When expressed on a mass basis, these values are numerically equivalent to mass percentages according to the relationship 1% (*w w*^−1^) = 10 mg g^−1^ EO.

### 3.7. Total Phenolic Content (TPC)

The TPC of sacha inchi oil was determined using the Folin–Ciocalteu assay, adapted from Saura-Calixto et al. [67]. First, gallic acid solutions (C_7_H_6_O_5_) were prepared at a concentration of 450 μg mL^−1^ (0.0225 g of C_7_H_6_O_5_ in 50 mL of distilled water), along with a 20% sodium carbonate (Na_2_CO_3_) solution (2 g of Na_2_CO_3_ in 10 mL of distilled water, sonicated for 5 min), and Folin–Ciocalteu reagent diluted to 2N. From the 450 μg mL^−1^ gallic acid stock solution, various concentrations were prepared to build the calibration curve (7.2, 14.4, 21.6, 28.8, and 36 μg mL^−1^). In Eppendorf tubes, volumes of 20, 40, 60, 80, and 100 μL of the standard solution were added, followed by 100 μL of 2N Folin–Ciocalteu reagent (allowed to react for 5 min). Then, 50 μL of 20% Na_2_CO_3_ solution was added to each tube, and distilled water was added to reach final volumes of 1100, 1080, 1060, 1040, and 1000 μL, respectively. After 1 h of reaction time, 200 μL from each tube was transferred to a 96-well microplate for absorbance reading at 739 nm using a multimode microplate reader (Synergy™ H1, BioTek Instruments, Inc., Winooski, VT, USA). The calibration curve was: Y = 0.0453X − 0.0326 (R^2^ = 0.994), where X represents µg mL^−1^ gallic acid solution and Y the absorbance.

To obtain the phenolic extracts from the samples of sacha inchi oil, an 80% methanol solution was used to solubilize the phenolic compounds present in the oil. In Eppendorf tubes, 100 μL of oil sample was mixed with 900 μL of 80% methanol, and vigorously vortexed to ensure homogenization. Then, 800 μL of the extract was transferred to a new Eppendorf tube, followed by the addition of 100 μL of 2N Folin–Ciocalteu reagent, and the mixture was left to stand at room temperature for 5 min. Subsequently, 50 μL of 20% Na_2_CO_3_ solution and 300 μL of distilled water were added. The mixture was incubated in the dark for 1 h, allowing the characteristic blue chromophore to develop via redox reaction. Finally, 200 μL of each sample was transferred to a 96-well microplate for absorbance measurement at 739 nm using the previously described reader. Results are expressed as milligrams of gallic acid equivalents (GAE) per 100 g of sample (mg GAE 100 g^−1^). Each analysis was performed in triplicate.

### 3.8. Antioxidant Capacity (AC)

The AC of the sacha inchi oil and the essential oils was determined using the DPPH (2,2-diphenyl-1-picrylhydrazyl) free radical scavenging assay, adapted from Kim et al. [68] with modifications for lipophilic matrices. For reagent preparation, a standard Trolox solution was prepared at 1 mM by dissolving 0.0125 g of Trolox in 50 mL of 80% methanol. Likewise, a 1 mM DPPH solution was prepared by dissolving 3.9 mg of DPPH in 100 mL of 80% methanol. This solution was stored in an amber glass bottle, homogenized by magnetic stirring for one hour (VELP Scientifica^®^, ARE, Usmate, Italy), and refrigerated at 5 °C until use. To construct the calibration curve, different concentrations of Trolox were prepared (500, 400, 200, 100, 50, 25, 10, and 5 μM), from which 10 μL aliquots were mixed with 190 μL of the DPPH solution. The mixtures were incubated in the dark for 10 min, and absorbance was measured at 515 nm using the previously mentioned microplate reader. The calibration curve was: Y = 0.044X + 1.983 (R^2^ = 0.9985), where X represents the Trolox concentration (μmol 100 g^−1^) and Y the DPPH radical inhibition percentage. For the oil sample analysis, 10 μL of the previously prepared phenolic extract was mixed with 190 μL of DPPH solution. After 10 min of incubation in the dark, absorbance was recorded at 515 nm. Results were expressed as micromoles of Trolox equivalents per gram of sample (μmol Trolox 100 g^−1^). All analyses were conducted in triplicate to ensure result reproducibility.

### 3.9. Oxidative Stability Index (OSI) Determination and Shelf-Life Estimation

The test was performed on an oxidative stability analyzer (Metrohm, 743 Rancimat, Herisau, Switzerland) to evaluate the OSI (hours) of the samples according to the AOCS Cd 12b-92 method. Rancimat parameters were programmed at three different reaction temperatures (90, 100 and 110 °C) with an air flow (15 L h^−1^) and constant sample weight (3.00 ± 0.1 g). The temperatures were selected according to the oxidation resistance of sacha inchi oil [59,69]. Three independent OSI determinations were performed at each temperature for each treatment.

The experimentally determined OSI values at 90, 100, and 110 °C were used to estimate the shelf-life of the samples at 25 °C by temperature extrapolation, following the approach proposed by Heidarpour and Farhoosh [70]. The relationship between OSI and temperature was described using the corresponding temperature-dependent equation:

Log (OSI) = α(T) + β

where α and β are constants. The resulting values represent estimated shelf-life under the model assumptions and should not be interpreted as experimentally verified shelf-life under real-time storage conditions.

### 3.10. Lipid Autoxidation Thermokinetics

The kinetic parameters of lipid autoxidation ($E_{a}\;$and $Q_{10}$) were determined from the OSI obtained at different temperatures (90, 100 and 110 °C). Three independent OSI determinations were performed at each temperature. For each independent replicate, the corresponding OSI values obtained at the three temperatures were used to perform an Arrhenius-type linear regression of Ln (OSI) versus 1/T, performed according to:

$$Ln\left(OSI\right)=b+m\left(\frac{1}{T}\right)$$

where T is the absolute temperature (K), b is the intercept, and m is the regression slope. The apparent activation energy (Ea, kJ mol^−1^) is calculated as:

Ea = mR

where *R* is the gas constant (8.314 J mol^−1^ K^−1^). Thus, three independent Ea values are obtained for each treatment and subsequently expressed as mean ± standard deviation. The coefficient of determination (R^2^) was used to assess the goodness of fit of each Arrhenius-type regression.

The temperature acceleration factor ($Q_{10}$) is calculated according to Farhoosh [61]. For each independent replicate, the corresponding OSI values are used to calculate $Q_{10}$:

$$Q_{10}=\;\frac{OSI\;at\;time\;T\;}{OSI\;at\;T\;+10\;{}^{\circ}C}$$

The resulting $Q_{10}\;$values are expressed as mean ± standard deviation for each treatment.

The thermodynamics parameters are calculated independently for each determination and subsequently expressed as mean ± standard deviation. The change in activation enthalpy (${\Delta H}^{++}$, kJ/mol) and change in activation entropy (${\Delta S}^{++}$, J mol^−1^ K^−1^) were obtained by regressing Log (k/T) versus (1/T), with the equation derived from the activated complex theory, according to Heidarpour and Farhoosh [70]:

$$Log\left(\frac{K}{T}\right)=\left[Log\left(\frac{k_{B}}{h}\right)+\left(\frac{∆S^{++}}{2.303R}\right)\right]-\left(\frac{∆H^{++}}{2.303R}\right)\left(\frac{1}{T}\right)$$

where: $k_{B}$ = 1.380658 × 10^−23^ J K^−1^ (Boltzmann constant), h = 6.6260755 × 10^−34^ Js (Planck’s constant) and K = 1/OSI. The resulting thermodynamic parameters are expressed as mean ± standard deviation for each treatment. Finally, the change in the Gibbs free energy (${\Delta G}^{++}$, kJ mol^−1^) is calculated according to equation: ${\Delta G}^{++}={\Delta H}^{++}-T{\Delta S}^{++}$.

### 3.11. Statistical Analysis

All analyses were performed using three independent experimental samples. A Completely Randomized Design (CRD) was employed for the experimental treatments. To compare oxidative stability, estimated shelf-life, and thermokinetic parameters among the oil samples, including the untreated control, the data were analyzed using one-way analysis of variance (ANOVA). Additionally, the estimated shelf-life data of the EO-treated samples were subjected to a three-way factorial ANOVA with a 3 × 3 × 5 factorial arrangement, considering essential oil (EO) type (matico, real lemon, and spearmint), EO concentration (0.1%, 0.3%, and 0.5%), and storage time (0, 7, 15, 30, and 90 days) as fixed factors. This factorial analysis was performed exclusively on the EO-treated samples; therefore, the untreated control was not included in this model because it did not have corresponding levels for the EO type and concentration factors. The factorial ANOVA evaluated the main effects of EO type, EO concentration, and storage time, as well as the two-way interactions (EO × concentration, EO × storage time, and concentration × storage time) and the three-way interaction (EO × concentration × storage time).

Before ANOVA, the normality of the data was verified and, when necessary, the data were logarithmically transformed to meet the assumptions of the analysis. When significant differences were detected, Tukey’s test was applied for multiple comparisons at a significance level of *p* < 0.05. For the factorial ANOVA, the magnitude of the main and interaction effects was assessed using partial eta squared (η^2^p). Data processing was performed using Minitab Statistical Software version 18 (Minitab, LLC, State College, PA, USA). Means and standard deviations were calculated using Microsoft Excel^®^ (Microsoft, Redmond, WA, USA).

## 4. Conclusions

The incorporation of essential oils (EOs) influenced the oxidative stability, thermokinetic behavior, and estimated shelf-life of sacha inchi oil. The magnitude of these effects depended on EO type, concentration, and storage time, although the factorial analysis indicated that these factors acted primarily as independent main effects under the experimental conditions evaluated. Spearmint EO showed the most persistent protective effect, whereas real lemon EO maintained relatively consistent protection during storage and matico EO showed a greater decline in protection over prolonged storage.

EO treatment increased the apparent energetic barrier to oxidative deterioration, as reflected by changes in apparent activation energy and related thermodynamic parameters. These parameters should be interpreted as apparent descriptors of the temperature dependence of the overall oxidation process rather than as direct evidence of specific molecular mechanisms. The combined evaluation of OSI, thermokinetic parameters, and estimated shelf-life provides a useful framework for comparing natural antioxidant treatments in highly unsaturated edible oils. However, the estimated shelf-life values obtained from accelerated oxidation experiments should not be considered experimentally validated shelf-life under real storage conditions. Therefore, further studies using real-time storage conditions are required to validate the predicted shelf-life and to monitor primary and secondary oxidation products, as well as relevant EO constituents, in order to better characterize the persistence and mechanisms of oxidative protection.

## Figures and Tables

**Table 1:** Characterization of sacha inchi oil.

Characteristics	Value ($\bar{X}\;\pm \;S$)
Extraction yield (%)	45.325 ± 2.213
Fatty acids	
C16:0	4.120 ± 0.020
C17:0	0.013 ± 0.006
C18:0	3.207 ± 0.031
C18:1	9.537 ± 0.032
C18:2 (ω-6)	35.267 ± 0.015
C18:3 (ω-3)	46.700 ± 0.046
C20:1 (ω-9)	0.217 ± 0.015
SFA	7.340 ± 0.026
PUFAS	81.967 ± 0.032
MUFAS	9.753 ± 0.035
Other fatty acids	0.940 ± 0.036
ω-6/ω-3	0.755 ± 0.001
PV (mEqO_2_ kg^−1^)	4.890 ± 0.040
AV (mg KOH g^−1^)	0.430 ± 0.010
Refraction Index	1.480 ± 0.000
IV (mg I_2_ g^−1^)	178.120 ± 1.120
p-Anisidine value	15.260 ± 0.550
TotOx	24.990 ± 0.130
TPC (mg GAE 100 g^−1^)	110.230 ± 1.234
AC (µmol Trolox g^−1^)	350.652 ± 1.760

SFA: Saturated Fatty Acids; PUFAS: Polyunsaturated Fatty Acids; MUFAS: Monounsaturated Fatty Acids; AV: Acid Value; IV: Iodine Value; PV: Peroxide Value; TPC: Total Phenolic Compounds; GAE: Gallic Acid Equivalent; AC: Antioxidant Capacity; $\bar{X}$: mean, S: standard deviation.

**Table 2:** Concentrations of selected volatile compounds in the essential oils (EOs).

EOs	Yield (%)	AC (µmol Trolox g^−1^)	Selected Predominant Volatile Compound	Characteristic Ion (*m*/*z*)	Retention Time (min)	Concentration (mg g^−1^ EO)	Compound Class
Matico	0.050 ± 0.005 ^c^	7023.188 ± 6.325 ^a^	Dillapiole (C_12_H_14_O_4_)	137	13.8	352.10 ± 0.22 ^c^	Phenylpropanoid
			Myristicin (C_11_H_12_O_3_)	151	10.2	145.10 ± 0.10 ^f^	Phenylpropanoid
Real lemon	0.530 ± 0.015 ^a^	2751.580 ± 3.432 ^b^	Limonene (C_10_H_16_)	68	6.5	428.42 ± 0.32 ^a^	Monoterpene hydrocarbon
			β-Pinene (C_10_H_16_)	93	5.9	183.07 ± 0.15 ^e^	Monoterpene hydrocarbon
Spearmint	0.130 ± 0.015 ^b^	777.429 ± 5.055 ^c^	Menthol (C_10_H_20_O)	71	11.4	376.50 ± 0.21 ^b^	Monoterpene alcohol
			Menthone (C_10_H_18_O)	112	10.1	219.46 ± 0.03 ^d^	Monoterpene ketone

AC: Antioxidant capacity, *m*/*z*: mass-to-charge ratio of the characteristic ion used for compound identification. Different letters in the same column (concentration) indicate significant differences (*p* < 0.05).

**Table 3:** Oxidative stability index (OSI) and estimated shelf-life (T = 25 °C) of sacha inchi oil with the addition of essential oils (EOs).

Temperature (°C)	EO	OSI (Hours)															
		Control	0.1%					0.3%					0.5%				
			0 d	7 d	15 d	30 d	90 d	0 d	7 d	15 d	30 d	90 d	0 d	7 d	15 d	30 d	90 d
90	Matico	6.717 ± 0.117 ^j^	8.250 ± 0.036 ^e^	8.070 ± 0.026 ^g^	7.810 ± 0.017 ^h^	7.720 ± 0.017 ^h^	7.350 ± 0.010 ^i^	8.777 ± 0.021 ^c^	8.777 ± 0.021 ^c^	8.210 ± 0.010 ^e,f^	8.120 ± 0.010 ^f,g^	7.750 ± 0.030 ^h^	9.160 ± 0.010 ^a^	8.940 ± 0.010 ^b^	8.580 ± 0.040 ^d^	8.487 ± 0.031 ^d^	8.123 ± 0.025 ^f,g^
	Real Lemon	6.717 ± 0.117 ^i^	8.413 ± 0.015 ^b,c,d^	8.370 ± 0.010 ^c,d,e^	8.263 ± 0.025 ^e,f,g^	8.160 ± 0.030 ^g^	7.743 ± 0.040 ^h^	8.530 ± 0.038 ^a,b^	8.443 ± 0.038 ^b,c^	8.327 ± 0.035 ^c,d,e,f^	8.220 ± 0.026 ^f,g^	7.803 ± 0.025 ^h^	8.613 ± 0.006 ^a^	8.500 ± 0.026 ^a,b^	8.407 ± 0.021 ^b,c,d^	8.290 ± 0.020 ^d,e,f^	7.830 ± 0.035 ^h^
	Spearmint	6.717 ± 0.117 ^j^	8.537 ± 0.015 ^b,c,d,e^	8.430 ± 0.010 ^d,e,f^	8.257 ± 0.025 ^f,g,h^	8.187 ± 0.030 ^h^	7.903 ± 0.040 ^i^	8.700 ± 0.038 ^a,b^	8.583 ± 0.038 ^b,c,d^	8.327 ± 0.035 ^f,g,h^	8.227 ± 0.026 ^g,h^	7.823 ± 0.025 ^i^	8.813 ± 0.006 ^a^	8.653 ± 0.026 ^a,b,c^	8.517 ± 0.021 ^c,d,e^	8.387 ± 0.020 ^e,f,g^	7.880 ± 0.035 ^i^
100	Matico	2.547 ± 0.099 ^j^	2.977 ± 0.015 ^e,f^	2.940 ± 0.026 ^f^	2.783 ± 0.015 ^h,i^	2.767 ± 0.012 ^h,i^	2.693 ± 0.015 ^i^	3.060 ± 0.030 ^e^	3.060 ± 0.030 ^e^	2.917 ± 0.015 ^f,g^	2.900 ± 0.010 ^f,g^	2.837 ± 0.021 ^g,h^	3.707 ± 0.029 ^a^	3.593 ± 0.015 ^b^	3.507 ± 0.015 ^b,c^	3.467 ± 0.015 ^c^	3.307 ± 0.023 ^d^
	Real Lemon	2.547 ± 0.099 ^f^	3.053 ± 0.015 ^a,b^	3.033 ± 0.006 ^a,b^	2.983 ± 0.015 ^b,c^	2.943 ± 0.015 ^c^	2.767 ± 0.012 ^e^	3.083 ± 0.021 ^a^	3.043 ± 0.021 ^a,b^	3.030 ± 0.010 ^a,b^	2.987 ± 0.012 ^b,c^	2.813 ± 0.021 ^d,e^	3.073 ± 0.006 ^a^	3.047 ± 0.015 ^a,b^	3.007 ± 0.015 ^a,b,c^	2.977 ± 0.012 ^b,c^	2.857 ± 0.025 ^d^
	Spearmint	2.547 ± 0.099 ^f^	3.093 ± 0.006 ^a,b,c^	3.060 ± 0.010 ^a,b,c^	3.030 ± 0.010 ^b,c,d^	3.017 ± 0.015 ^c,d^	2.957 ± 0.042 ^d,e^	3.090 ± 0.012 ^a,b,c^	3.063 ± 0.012 ^a,b,c^	3.037 ± 0.012 ^b,c,d^	3.010 ± 0.010 ^c,d^	2.897 ± 0.012 ^e^	3.130 ± 0.020 ^a^	3.110 ± 0.010 ^a,b^	3.080 ± 0.010 ^a,b,c^	3.043 ± 0.006 ^a,b,c,d^	2.893 ± 0.032 ^e^
110	Matico	0.947 ± 0.023 ^g^	1.037 ± 0.012 ^d,e^	1.013 ± 0.015 ^d,e,f^	0.980 ± 0.010 ^e,f,g^	0.970 ± 0.010 ^f,g^	0.947 ± 0.006 ^g^	1.100 ± 0.010 ^a,b,c^	1.100 ± 0.010 ^a,b,c^	1.050 ± 0.050 ^c,d^	1.040 ± 0.040 ^c,d,e^	0.983 ± 0.006 ^e,f,g^	1.150 ± 0.010 ^a^	1.117 ± 0.015 ^a,b^	1.063 ± 0.025 ^b,c,d^	1.057 ± 0.021 ^b,c,d^	1.030 ± 0.010 ^d,e,f^
	Real Lemon	0.947 ± 0.023 ^g^	1.060 ± 0.010 ^a^	1.053 ± 0.006 ^a,b^	1.037 ± 0.010 ^a,b,c^	1.027 ± 0.006 ^b,c,d^	0.997 ± 0.012 ^d,e,f^	1.067 ± 0.006 ^a^	1.053 ± 0.012 ^a,b^	1.037 ± 0.006 ^a,b,c^	1.027 ± 0.006 ^b,c,d^	0.993 ± 0.006 ^e,f^	1.057 ± 0.006 ^a,b^	1.040 ± 0.010 ^a,b,c^	1.027 ± 0.015 ^b,c,d^	1.020 ± 0.010 ^c,d,e^	0.983 ± 0.015 ^f^
	Spearmint	0.947 ± 0.023 ^f^	1.077 ± 0.006 ^a,b^	1.060 ± 0.010 ^a,b,c^	1.033 ± 0.010 ^c,d^	1.030 ± 0.010 ^c,d^	1.013 ± 0.015 ^d,e^	1.090 ± 0.010 ^a^	1.063 ± 0.015 ^a,b,c^	1.040 ± 0.010 ^b,c,d^	1.033 ± 0.006 ^c,d^	1.003 ± 0.006 ^d,e^	1.083 ± 0.006 ^a^	1.060 ± 0.010 ^a,b,c^	1.040 ± 0.010 ^b,c,d^	1.027 ± 0.012 ^c,d^	0.987 ± 0.025 ^e^
Shelf-Life (d)	Matico	164.9 ± 25.3 ^e^	293.0 ± 14.7 ^a,b,c,d^	288.3 ± 14.2 ^a,b,c,d^	277.4 ± 7.9 ^a,b,c,d^	269.9 ± 7.0 ^c,d^	240.9 ± 4.0 ^d^	310.7 ± 10.0 ^a,b,c^	310.7 ± 10.0 ^a,b,c^	276.1 ± 45.8 ^b,c,d^	273.9 ± 37.4 ^b,c,d^	267.3 ± 3.4 ^c,d^	338.8 ± 9.3 ^a^	336.0 ± 17.9 ^a,b^	333.8 ± 29.8 ^a,b^	324.8 ± 22.3 ^a,b,c^	291.2 ± 12.8 ^d^
	Real Lemon	164.9 ± 25.3 ^g^	296.6 ± 12.1 ^a,b,c,d,e^	295.9 ± 5.5 ^a,b,c,d,e^	295.0 ± 9.0 ^b,c,d,e^	286.2 ± 9.0 ^d,e,f^	252.4 ± 10.6 ^f^	308.0 ± 8.0 ^a,b,c,d^	307.2 ± 15.2 ^a,b,c,d^	305.9 ± 8.3 ^a,b,c,d^	297.5 ± 7.1 ^a,b,c,d,e^	264.9 ± 8.3 ^e,f^	330.5 ± 5.7 ^a^	329.7 ± 8.1 ^a,b^	328.1 ± 15.4 ^a,b^	318.0 ± 9.7 ^a,b^	279.6 ± 11.0 ^d,e,f^
	Spearmint	164.9 ± 25.3 ^e^	299.6 ± 5.6 ^a,b,c,d^	299.1 ± 5.8 ^a,b,c,d^	298.7 ± 3.1 ^a,b,c,d^	291.8 ± 4.7 ^a,b,c,d^	265.2 ± 11.3 ^c,d^	310.3 ± 9.4 ^a,b,c^	320.1 ± 40.3 ^a,b^	302.9 ± 12.1 ^a,b,c,d^	294.2 ± 8.0 ^a,b,c,d^	261.3 ± 11.1 ^d^	335.4 ± 8.8 ^a^	334.6 ± 9.4 ^a^	333.7 ± 14.6 ^a^	323.7 ± 12.7 ^a,b^	285.2 ± 17.8 ^b,c,d^

Identical letters in the same row do not show significant differences in OSI values (*p* < 0.05); EOs: essential oils; d: days.

**Table 4:** Three-way factorial ANOVA of estimated shelf-life of sacha inchi oil treated with essential oils.

Source of Variation	DF	SS	MS	F	*p*-Value	η^2^p
Essential oil (EO)	2	1513.092	756.546	3.146	0.0478	0.0653
EO concentration	2	34,946.068	17,473.034	72.661	<0.001	0.6175
Storage time	4	39,388.989	9847.247	40.949	<0.001	0.6454
EO × concentration	4	2109.344	527.336	2.193	0.0761	0.0888
EO × storage time	8	1244.875	155.609	0.647	0.7361	0.0544
Concentration × storage time	8	1173.899	146.737	0.610	0.7671	0.0514
EO × concentration × storage time	16	1268.769	79.298	0.330	0.9925	0.0554
Error	90	21,642.640	240.474	—	—	—
Total	134	103,287.676	—	—	—	—

DF, degrees of freedom; SS, sum of squares; MS, mean square; F, F-statistic; *p*, probability value; η^2^p, partial eta squared. The analysis was performed using a 3 × 3 × 5 factorial arrangement, with essential oil type (matico, real lemon, and spearmint), EO concentration (0.1%, 0.3%, and 0.5%), and storage time (0, 7, 15, 30, and 90 days) as fixed factors. The analysis included all main effects, two-way interactions, and the three-way interaction. Statistical significance was considered at *p* < 0.05.

**Table 5:** Thermokinetic parameters of sacha inchi oil with the addition of essential oils (EOs).

Parameters	EOs	Control	0.1%					0.3%					0.5%				
			0 d	7 d	15 d	30 d	90 d	0 d	7 d	15 d	30 d	90 d	0 d	7 d	15 d	30 d	90 d
Ea (kJ mol^−1^)	Matico	113.22 ± 2.43 ^i^	119.84 ± 0.84 ^e^	119.87 ± 0.89 ^e^	119.93 ± 0.47 ^d,e^	119.85 ± 0.48 ^d,e^	118.41 ± 0.28 ^f^	120.02 ± 0.58 ^c,d^	120.02 ± 0.58 ^c,d^	118.88 ± 2.81 ^f^	118.78 ± 2.24 ^f^	119.26 ± 0.17 ^e,f^	119.77 ± 0.47 ^d,e^	120.07 ± 0.84 ^c,d^	120.51 ± 1.49 ^b,c^	120.24 ± 1.23 ^c^	119.20 ± 0.67 ^e,f^
	Real Lemon	113.22 ± 2.43 ^i^	119.68 ± 0.64 ^c,d^	119.74 ± 0.32 ^d^	119.93 ± 0.49 ^c,d^	119.76 ± 0.52 ^c,d^	118.47 ± 0.69 ^e^	120.11 ± 0.39 ^c^	120.25 ± 0.76 ^c^	120.36 ± 0.42 ^c^	120.17 ± 0.42 ^c^	119.09 ± 0.50 ^d,e^	121.22 ± 0.30 ^a^	121.37 ± 0.43 ^a^	121.48 ± 0.78 ^a^	121.05 ± 0.51 ^a,b^	119.86 ± 0.64 ^d,e^
	Spearmint	113.22 ± 2.43 ^j^	119.62 ± 0.30 ^d^	119.79 ± 0.38 ^d^	120.05 ± 0.18 ^c,d^	119.74 ± 0.41 ^d^	118.65 ± 0.66 ^e^	120.02 ± 0.49 ^c^	120.66 ± 1.88 ^b^	120.17 ± 0.64 ^c^	119.84 ± 0.50 ^c,d^	118.64 ± 0.73 ^e^	121.11 ± 0.43 ^a^	121.30 ± 0.46 ^a^	121.47 ± 0.69 ^a^	121.33 ± 0.68 ^a^	120.03 ± 1.12 ^c^
$Q_{10}$	Matico	2.665 ± 0.100 ^d^	2.822 ± 0.059 ^b^	2.823 ± 0.088 ^b^	2.823 ± 0.035 ^b^	2.821 ± 0.044 ^b^	2.787 ± 0.066 ^c^	2.825 ± 0.055 ^b^	2.825 ± 0.055 ^b^	2.798 ± 0.078 ^c^	2.795 ± 0.062 ^c^	2.809 ± 0.088 ^b,c^	2.847 ± 0.413 ^a^	2.853 ± 0.402 ^a^	2.873 ± 0.469 ^a^	2.865 ± 0.458 ^a^	2.834 ± 0.414 ^b^
	Real Lemon	2.665 ± 0.100 ^d^	2.818 ± 0.073 ^b^	2.820 ± 0.066 ^b^	2.824 ± 0.062 ^b^	2.820 ± 0.055 ^b^	2.788 ± 0.032 ^c^	2.829 ± 0.070 ^b^	2.832 ± 0.069 ^b^	2.836 ± 0.098 ^b^	2.831 ± 0.088 ^b^	2.803 ± 0.037 ^c^	2.856 ± 0.059 ^a^	2.860 ± 0.081 ^a^	2.863 ± 0.081 ^a^	2.852 ± 0.076 ^a^	2.823 ± 0.103 ^b^
	Spearmint	2.665 ± 0.100 ^d^	2.816 ± 0.064 ^b^	2.821 ± 0.073 ^b^	2.829 ± 0.115 ^b^	2.821 ± 0.121 ^b^	2.796 ± 0.147 ^c^	2.825 ± 0.026 ^b^	2.842 ± 0.065 ^b^	2.831 ± 0.101 ^b^	2.823 ± 0.100 ^b^	2.794 ± 0.107 ^c^	2.853 ± 0.046 ^a^	2.858 ± 0.086 ^a^	2.863 ± 0.110 ^a^	2.860 ± 0.117 ^a^	2.828 ± 0.121 ^b^
${\Delta H}^{++}$(kJ mol^−1^)	Matico	110.14 ± 2.43 ^h^	116.76 ± 0.84 ^c^	116.79 ± 0.89 ^c^	116.85 ± 0.47 ^c^	116.77 ± 0.48 ^c^	115.33 ± 0.28 ^g^	116.94 ± 0.58 ^c^	116.94 ± 0.58 ^c^	115.81 ± 2.81 ^g^	115.70 ± 2.25 ^g^	116.19 ± 0.17 ^f^	116.69 ± 0.47 ^c^	116.99 ± 0.84 ^c^	117.43 ± 1.49 ^b,c^	117.16 ± 1.23 ^b,c^	116.12 ± 0.67 ^f^
	Real Lemon	110.14 ± 2.43 ^h^	116.60 ± 0.64 ^c,d^	116.67 ± 0.32 ^c,d^	116.85 ± 0.49 ^c^	116.68 ± 0.52 ^c,d^	115.39 ± 0.69 ^g^	117.03 ± 0.39 ^c^	117.17 ± 0.76 ^b,c^	117.28 ± 0.42 ^b,c^	117.10 ± 0.42 ^b,c^	116.02 ± 0.50 ^f^	118.14 ± 0.30 ^a^	118.29 ± 0.43 ^a^	118.40 ± 0.78 ^a^	117.97 ± 0.51 ^a,b^	116.78 ± 0.64 ^e^
	Spearmint	110.14 ± 2.43 ^h^	116.54 ± 0.30 ^d^	116.71 ± 0.38 ^c,d^	116.97 ± 0.18 ^c^	116.66 ± 0.41 ^c,d^	115.57 ± 0.66 ^g^	116.94 ± 0.49 ^c^	117.58 ± 1.88 ^b^	117.09 ± 0.64 ^b,c^	116.76 ± 0.50 ^c^	115.56 ± 0.73 ^g^	118.03 ± 0.43 ^a^	118.22 ± 0.46 ^a^	118.39 ± 0.69 ^a^	118.25 ± 0.68 ^a^	116.95 ± 1.12 ^e^
${\Delta S}^{++}$(J mol^−1^ K^−1^)	Matico	40.87 ± 6.60 ^f^	57.38 ± 2.29 ^b^	57.62 ± 2.45 ^b^	58.12 ± 1.29 ^b^	57.98 ± 1.31 ^b^	54.39 ± 0.78 ^c^	57.46 ± 1.59 ^b^	57.46 ± 1.59 ^b^	54.86 ± 7.67 ^c^	54.65 ± 6.14 ^c^	56.30 ± 0.46 ^b^	56.01 ± 1.29 ^b^	57.05 ± 2.29 ^b^	58.55 ± 4.05 ^b^	57.90 ± 3.35 ^b^	55.43 ± 1.81 ^c^
	Real Lemon	40.87 ± 6.60 ^f^	56.77 ± 1.73 ^b^	56.99 ± 0.89 ^b^	57.61 ± 1.32 ^b^	57.27 ± 1.41 ^b^	54.20 ± 1.87 ^c^	57.84 ± 1.04 ^b^	58.32 ± 2.06 ^b^	58.70 ± 1.12 ^b^	58.31 ± 1.12 ^b^	55.81 ± 1.34 ^c^	60.83 ± 0.82 ^a^	61.33 ± 1.18 ^a^	61.73 ± 2.13 ^a^	60.66 ± 1.39 ^a^	57.85 ± 1.75 ^b^
	Spearmint	40.87 ± 6.60 ^f^	56.49 ± 0.81 ^b^	57.06 ± 1.05 ^b^	57.90 ± 0.49 ^b^	57.13 ± 1.13 ^b^	54.39 ± 1.79 ^c^	57.48 ± 1.34 ^b^	59.31 ± 5.03 ^b^	58.18 ± 1.73 ^b^	57.38 ± 1.34 ^b^	54.49 ± 2.01 ^c^	60.36 ± 1.17 ^a^	60.99 ± 1.25 ^a^	61.57 ± 1.86 ^a^	61.30 ± 1.85 ^a^	58.24 ± 3.10 ^b^
${\Delta G}^{++}$ (kJ mol^−1^)	Matico	94.89 ± 0.34 ^f^	95.36 ± 0.59 ^d^	95.29 ± 0.60 ^d^	95.18 ± 0.57 ^e^	95.15 ± 0.57 ^e^	95.04 ± 0.54 ^e^	95.52 ± 0.56 ^c^	95.52 ± 0.56 ^c^	95.37 ± 0.49 ^d^	95.34 ± 0.50 ^d^	95.19 ± 0.55 ^e^	95.81 ± 0.55 ^a^	95.70 ± 0.59 ^b^	95.61 ± 0.60 ^b^	95.57 ± 0.59 ^b^	95.43 ± 0.56 ^c^
	Real Lemon	94.89 ± 0.34 ^f^	95.42 ± 0.58 ^d^	95.41 ± 0.58 ^d^	95.35 ± 0.58 ^d^	95.31 ± 0.58 ^d^	95.17 ± 0.55 ^e^	95.45 ± 0.58 ^c^	95.42 ± 0.56 ^c^	95.39 ± 0.58 ^c^	95.35 ± 0.58 ^c^	95.19 ± 0.56 ^e^	95.45 ± 0.60 ^b^	95.42 ± 0.62 ^b^	95.39 ± 0.61 ^b^	95.35 ± 0.60 ^b^	95.19 ± 0.59 ^e^
	Spearmint	94.89 ± 0.34 ^f^	95.47 ± 0.56 ^d^	95.43 ± 0.59 ^d^	95.37 ± 0.58 ^d^	95.35 ± 0.58 ^d^	95.27 ± 0.55 ^e^	95.51 ± 0.57 ^c^	95.43 ± 0.60 ^c^	95.39 ± 0.59 ^c^	95.36 ± 0.58 ^c^	95.23 ± 0.54 ^e^	95.53 ± 0.60 ^b^	95.47 ± 0.61 ^b^	95.42 ± 0.61 ^b^	95.37 ± 0.62 ^b^	95.21 ± 0.63 ^e^

Identical letters in the same row do not show significant differences in thermokinetic parameters values (*p* < 0.05); d: days; activation energy (Ea); temperature acceleration factor (Q_10_); enthalpy (${\Delta H}^{++}$); entropy (${\Delta S}^{++}$); Gibbs free energy (${\Delta G}^{++}$); Δ denotes the change in the corresponding thermodynamic parameter.

## Data Availability

All the data related to this work are given in the manuscript.

## Abbreviations

EOs	Essential Oils
OSI	Oxidative Stability Index
Ea	Activation Energy
ΔH^++^	Change in the Activation Enthalpy
ΔS^++^	Change in the Activation Entropy
ΔG^++^	Change in the Gibbs Free Energy
